# Low resistance to chytridiomycosis in direct-developing amphibians

**DOI:** 10.1038/s41598-017-16425-y

**Published:** 2017-11-30

**Authors:** Andréa F. C. Mesquita, Carolina Lambertini, Mariana Lyra, Leo R. Malagoli, Timothy Y. James, Luís Felipe Toledo, Célio F. B. Haddad, C. Guilherme Becker

**Affiliations:** 1 Universidade Estadual Paulista (UNESP), Instituto de Biociências, Departamento de Zoologia, and Centro de Aquicultura (CAUNESP), 13506-900 Rio Claro, SP Brazil; 2 Departamento de Biologia Animal, I.B., Universidade Estadual de Campinas, 13083-862 Campinas, SP Brazil; 3 Department of Ecology and Evolutionary Biology, University of Michigan, 48109 Ann Arbor, MI USA; 4 Department of Biological Sciences, The University of Alabama, 35487 Tuscaloosa, AL USA

## Abstract

Host-generalist pathogens sporadically infect naive hosts, potentially triggering epizootics. The waterborne fungus *Batrachochytrium dendrobatidis* (*Bd*) is linked to declines of hundreds of amphibian species with aquatic larvae. Although several population declines and extinctions attributed to *Bd* have been reported among cryptic species undergoing direct development away from water, epidemiological studies focused on these terrestrial frogs are lacking. Our field data support that terrestrial direct-developing hosts are less exposed to *Bd* during their ontogeny than species with aquatic larvae, and thus they might lack adaptive responses against waterborne chytrids. Using controlled laboratory experiments, we exposed wild-caught amphibian species with terrestrial and aquatic life histories to *Bd* and found that direct developers showed more rapid increases in infection loads and experienced higher mortality rates than species with aquatic larvae. Our findings provide novel information about host responses to generalist pathogens and specifically show that our focal direct developing species have low resistance to *Bd* infections. Finally, our results underscore that we should not ignore *Bd* as a potential threat to direct developing species simply because they are less exposed to *Bd* in nature; instead future amphibian conservation plans should include efforts to safeguard hundreds of direct-developing amphibian species globally.

## Introduction

The evolution of host responses to pathogens involves tradeoffs between the degree of exposure and the development of host defenses such as resistance to pathogen growth and tolerance of pathogen damage^1,2^. Thus, host life history traits are expected to play a major role in disease dynamics because host exposure to pathogens could be strongly influenced by factors such as habitat use and breeding strategy^3,4^. Host species that are frequently exposed to a given pathogen in environmental reservoirs or breeding habitats should evolve resistance and/or tolerance to this pathogen to some degree. Conversely, species that are only sporadically infected through the environment or spillover should develop minimal adaptations for pathogen resistance and/or tolerance.

The frog killing-fungus *Batrachochytrium dendrobatidis* (*Bd*), which is linked to amphibian declines globally^5^, is a host-generalist pathogen able to infect amphibian species with varying life histories^6^. *Bd* has a biphasic life-cycle with flagellated and free-swimming zoospores (often discharged into water bodies such as streams and ponds), and sessile zoosporangia, which attacks keratinized tissues of both tadpoles and post-metamorphic anurans^7–9^. Because *Bd* is a waterborne pathogen, amphibian species with aquatic larval development are frequently exposed to the pathogen in the natural environment^9^. Hundreds of montane stream-breeding frog species have experienced population declines and extinctions associated to *Bd* in both temperate and tropical regions^10–12^. Terrestrial amphibian species undergoing direct development in the forest leaf-litter or in underground retreats, however, only sporadically disperse through aquatic habitats such as streams and temporary ponds. Despite the reduced likelihood of environmental exposure to *Bd* within this group of frogs, there are dozens of records of population declines and extinctions attributed to chytridiomycosis among cryptic direct developing species from pristine environments^12–15^. The lower pathogen encounters in water bodies among direct-developing amphibians likely reduces exposure from repeated infections, which by itself bodes well in terms of conservation. However, lower exposure to *Bd* in water bodies might in turn preclude direct developing frogs from developing resistance or tolerance to *Bd* ^4,16^.

Here we tested (i) whether amphibian species with direct development and aquatic larvae show contrasting prevalence in the natural environment and (ii) whether different exposure levels to the pathogen in nature impact host resistance and tolerance as measured by frogs experimentally inoculated with *Bd* in the laboratory. Because frequent exposure to the waterborne *Bd* in the wild leads to adaptive responses in amphibians with aquatic larvae, we predicted lower risk of chytridiomycosis (higher resistance and/or tolerance) in our focal species with aquatic larvae experimentally exposed to *Bd*. We predicted that this mechanism would also lead to higher infection loads and *Bd*-associated mortality in our focal direct-developing amphibian species inoculated with the fungal pathogen.

## Results

**Figure 1:**
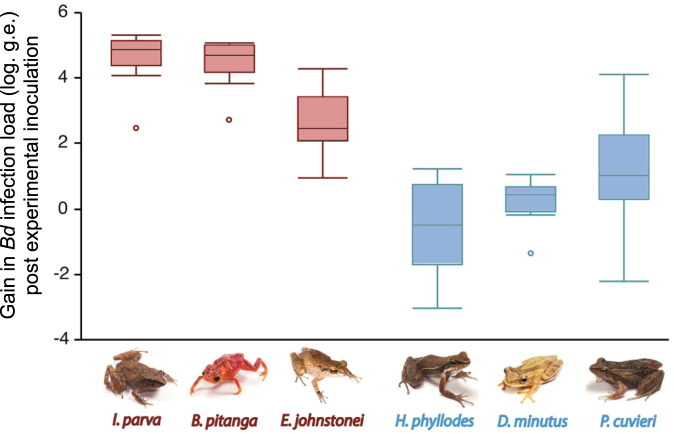
Gain in *Bd* infection load post experimental inoculation (difference between swab #2 log g.e. and swab #1 log g.e.) among amphibian species with direct development (shown in red) and aquatic larvae (shown in blue). The box delimits the first and third quartile, horizontal lines represents the median, and vertical lines delimit the maximum and minimum values, except for outliers that are represented by circles.

At the moment of capture in nature, direct-developing amphibian species showed lower *Bd* infection prevalence (*Ischnocnema parva*: 24%; *Brachycephalus pitanga*: 0%; *Eleutherodactylus johnstonei*: 0%) than species with aquatic larvae (*Dendropsophus minutus*: 30%; *Physalaemus cuvieri*: 100%; *Hylodes phyllodes*: 55%; *χ* ^2^ = 38.105, d.f. = 1, *P* < 0.0001). Following experimental inoculation of both direct-developing species and those with aquatic larvae, we found that direct-developing species exposed to *Bd* showed a higher gain in *Bd* infection loads when compared to species with aquatic larvae (*F*_[*1*, *3*.*9*]_ = 18.459, *P* < 0.013; Fig. 1; Table S1).

**Figure 2:**
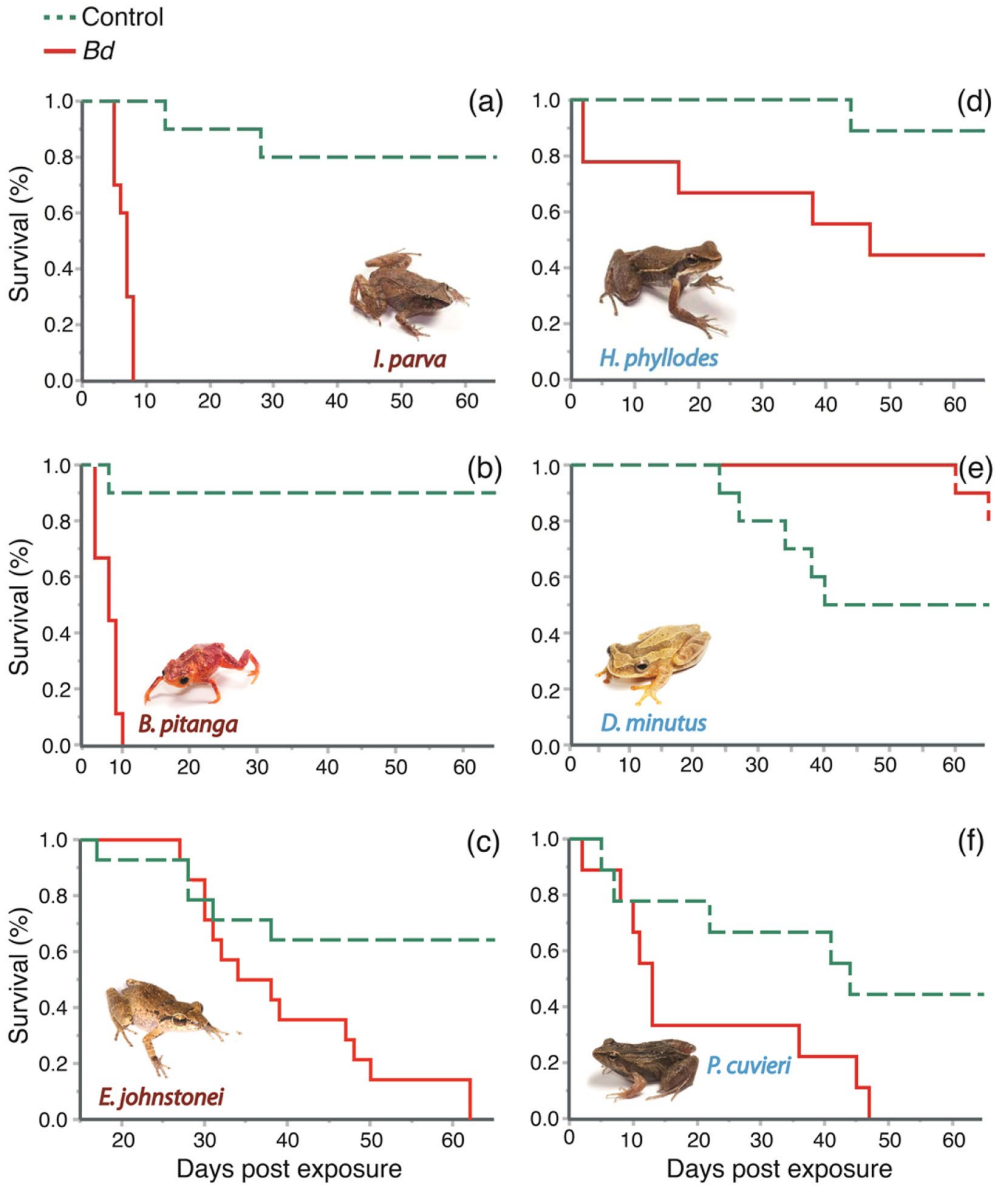
Survival curves for amphibian species with direct development (**A**–**C**) and aquatic larvae (**D**–**F**). *Bd*-inoculated and control frogs highlighted in solid red and dashed green lines, respectively.

Besides showing a higher gain in *Bd* infection loads post experimental inoculation, all three direct-developing amphibian species experienced higher mortality rates in the *Bd*-inoculation treatment than in the control treatment (*I*. *parva: χ* ^2^ = 17.561, df = 1, *P* < 0.001; *B*. *pitanga: χ* ^2^ = 12.929, df = 1, *P* < 0.001; *E*. *johnstonei: χ* ^2^ = 4.106; df = 1; *P* =  0.042, Fig. 2a–c). Conversely, two out of our three focal species with aquatic larvae showed similar survival rates among *Bd*-inoculated and control treatments (*D*. *minutus*: *χ* ^2^ = 3.232, df = 1, *P* = 0.072; *P*. *cuvieri: χ* ^2^ = 2.447, df = 1, *P* = 0.118; *H*. *phyllodes: χ* ^2^ = 4.290; df = 1, *P* = 0.038, Fig. 2d–f).

Our parametric survival model showed that host life history, species, and gain in *Bd* load were strong predictors of host survival, though the interaction between host life history and gain in *Bd* load was not a significant predictor (Table 1).

**Table 1:** Parametric survival model testing the effect of host life history, species, and gain in *Bd* load on host survival. One-level interaction between life history and gain in *Bd* load also included in the model. Significant variables are highlighted with an asterisk.

Parameter	d.f.	Chi Square	*P*
Life history	1	4.755	0.029*
Gain in *Bd* load	1	10.152	0.001*
Life history × Gain in *Bd* load	1	0.318	0.573
Species (residuals)	1	28.094	<0.0001*

Whole model statistics: Chi Square = 134.170, d.f. = 4, *P* < 0.0001.

## Discussion

Many conspicuous amphibian die-offs attributed to *Bd* have been documented along water bodies in tropical and temperate regions, with dead frogs spotted at the edge of streams and ponds. The fact that these declines were often caught on camera in dramatic pictures, coupled with the known waterborne cycle of *Bd*, contributed to the disproportionate number of experimental studies of chytridiomycosis focused on frogs with aquatic life-cycles. Nonetheless, population declines attributed to *Bd* also occurred in dozens of direct-developing amphibian species in the Neotropics^12–14^ and Africa^15^, but scientists failed to record die-offs in cryptic terrestrial environments. Our findings show that our three focal direct-developing species are highly susceptible to chytridiomycosis, likely due to the lack of adaptive responses to waterborne chytrids. Low exposure to *Bd* in the natural environment could certainly bode well for the conservation of direct developers. However, the observed high mortalities in experimental infections added to the fact that most direct-developing species may be already threatened due to their exceptionally narrow geographic ranges^17^ and the accelerated pace of habitat loss highlights the need to understand how biotic and abiotic factors interact and impact *Bd* transmission dynamics in this diverse group of frogs.

Host populations evolve responses to pathogens in the presence of selective forces such as frequent pathogen exposure^1^. Differences in host life history such as habitat use and reproduction may thus alter both host resistance and tolerance^18–21^. Lower encounter rates with water bodies throughout the ontogeny of direct developers is thus a candidate mechanism leading to a lack of strong host responses against *Bd*. In agreement with this hypothesis, our focal direct developing frogs showed (i) lower *Bd* infection prevalence at the moment of capture in the field, (ii) higher gains in *Bd* loads during the experiment, and (iii) higher mortality rates post experimental inoculation. These findings, combined, suggest that direct-developing species are less resistant to infections. However, our parametric survival analysis showed that the interaction between gain in *Bd* load during the experiment and host life history was not a good predictor of host survival; this suggests that although direct-developers are less resistant, we found no evidence that they are less tolerant to *Bd* infections.

Field studies also support higher prevalence among host species with aquatic larvae, though direct-developing species often carry much higher average infection loads^22,23^, suggesting that direct-developing frogs are more easily wiped out of the population if exposed to *Bd*. In other words, the likelihood of capturing an infected direct developer in nature may be small because chytridiomycosis may quickly lead to host mortality. In agreement with this prediction, two individuals of *I*. *parva* that tested positive at the moment of capture were assigned to the control treatment, but died with very high *Bd* infection loads (2400 and >200000 log G.E.). This shows that natural *Bd* infections could indeed lead to host mortality among direct-developing species. A rapid progress of disease in a small subsample of frogs from natural populations could thus preclude the development of acquired resistance and/or tolerance at the population level.

Several other mechanisms may also contribute to the observed higher mortality rates of direct-developing species. Species in the Neotropical direct development clade Brachycephaloidea, for instance, have in average much smaller geographic ranges than hylids and leptodactylids^17^, and smaller geographic range distributions may be linked to lower adaptive immunity to pathogens^24^. Animals with larger range distributions tend to be exposed to selective pressures from a myriad of pathogens and strains, which may in turn increase host adaptive defenses. Another possibility is that host genetics may directly impact the observed association between host life history and mortality. Genetic diversity within a species often decreases in populations negatively impacted by habitat alteration^25,26^. Direct-developing frogs show high philopatry to natural habitats and do not engage in extensive breeding migrations such as the ones carried out by frogs undergoing aquatic larval development^27,28^. This makes direct-developing species breeding in the forest leaf litter more prone to suffer from population isolation and inbreeding depression^27^. Lower adaptive potential of genetically depauperate populations or species^29^ may be thus linked to higher susceptibility to disease^30,31^. Population isolation may also impact critical components of host immunogenetic diversity such as genes of the major histocompatibility complex (MHC), which encode key regulators of acquired immune function. In particular, class II MHC gene diversity has been linked to pathogen resistance in a number of wildlife systems, including amphibians^26,32–34^. Nevertheless, two of our focal direct-developing species are not limited in gene flow because our collections were concentrated on continuous natural forest, and we think that strong inbreeding depression and genetic erosion is unlikely under these circumstances^35^.

Narrow geographic range distribution is also linked to higher local population densities in tropical frogs^36^; this could facilitate direct transmission among direct-developing species in the natural environment. The pumpkin toadlet *Brachycephalus pitanga*, for instance, is only known from PESM Núcleo Santa Virgínia, a protected area, and immediate surroundings, occurring at incredibly high population densities in many areas within its small range. Although there is no evidence of ongoing direct transmission in nature for our focal direct-developing species, *Bd* could potentially threaten these populations/species in the event of increased direct transmission, spillover, and/or enhanced exposure to *Bd* in the natural environment. A previous study conducted in the same protected area of Núcleo Santa Virgínia showed that several individuals of the congeneric *Ischnocnema guentheri* carried high natural infection loads (>10,000 zoospores)^22^. Likewise, direct-developing *Arthroleptis* were among the most impacted by the emergence of *Bd* in Cameroon, and a retrospective study detected high average infection loads in species of *Arthroleptis* at the onset of population declines^15^. We speculate that periods of extreme drought and heat may direct frogs to ravines or streambeds due to mild and humid microclimates. Thus, climate change could potentially trigger epizootics in populations of direct developers as previously postulated for species with aquatic larvae^12,37^.

The risk of population declines of direct developing frogs depends on the tradeoff between the level of host exposure and acquired resistance or tolerance in natural populations. At this point, the low *Bd* prevalence in our focal species in nature is likely contributing to population persistence. However, recent capture-recapture models support that cryptic disease-induced mortality might lead to host extinction in apparently stable populations of amphibians undergoing metamorphosis away from water bodies such as the Darwin frog *Rhinoderma darwinii* ^38^. This recent study suggests that *Bd*-induced extinctions could potentially occur even in the absence of epizootics and in scenarios of low pathogen prevalence. If environmental change facilitates spillover, direct transmission and/or environmental exposure to the pathogen, then low acquired resistance might become a threat to hundreds of direct developing species. Both habitat alteration and climate change are linked to shifts in host-pathogen dynamics in amphibians, thus, we speculate that these anthropogenic forces could impact direct-developing amphibian species from areas of enzootic chytridiomycosis such as the Neotropics and west Africa^14,15,39–42^. In Brazil, the direct-developing Itatiaia highland frog *Holoaden bradei* (Supplementary Photo S1) went extinct in the wild during a period of *Bd* upsurge in that area^12^. These findings, combined, underscore that further research is critical to safeguard direct-developing amphibian species globally.

## Methods

### Host Species

We captured adult anurans of five locally-abundant species in São Luiz do Paraitinga (−23.88°S, −46.56°W, 929.9 ± 71.4 m sd), State of São Paulo, in December 2015. We collected individuals from three species undergoing aquatic larval development (*Dendropsophus minutus*, Hylidae; *Physalaemus cuvieri*, Leptodactylidae; *Hylodes phyllodes*, Hylodidae). We also collected individuals from two species exhibiting terrestrial direct development (*Brachycephalus pitanga* and *Ischnocnema parva*, Brachycephalidae). We included an additional direct-developing species in our study; the Antillean *Eleutherodactylus johnstonei*, Eleutherodactylidae, from a recently introduced population in the city of São Paulo^43^. We also increased our sample size with a couple of individuals of *P*. *cuvieri* collected in the municipality of Rio Claro, also in the state of São Paulo. Terrestrial amphibian species had an average snout-vent length of 15.2 mm ± 4.1 sd and aquatic species 26.3 mm ± 1.3 sd. We brought 20 individuals of each amphibian species to the laboratory and conducted the experimental infection at Universidade Estadual Paulista (UNESP), Rio Claro, SP, Brazil.

### Bd cultures in the laboratory

We maintained cryopreserved cultures of *Bd* in the laboratory at Universidade Estadual de Campinas – UNICAMP, Brazil. Prior to the experimental infections, we thawed and cultured a local strain of *B*. *dendrobatidis* (GPL-1; CLFT159) in Petri plates with tryptone-agar 1% under the constant temperature of ~19 °C, for 7 days. At the first day of experiment (day zero), when we exposed animals to *Bd*, we filled each Petri plate with 5 ml of distilled water and waited for 20 minutes, scraping the substrate with a sterile scalpel to facilitate zoospore release. Then, we used a sterile syringe to strain the inoculum into a beaker, from which 1 ml was sampled to quantify zoospore concentration using a hemocytometer. Finally, we used a solution containing 1 × 10^6^ free-swimming zoospores per ml in experimental infections.

### Experimental design

We kept animals individually in plastic terraria (21 × 14 × 8 cm). We tilted aquaterraria on a 5-degree angle to produce both dry and wet environmental conditions. We covered one side of each aquaterrarium with autoclaved *Sphagnum* and the other side with 10 ml of distilled water. We used 10 individual frogs per species for each of the two following treatments: (i) *Bd*-inoculated – each individual frog was kept in a Petri plate in direct contact with 10 ml of *Bd* inoculum (total zoospore count 1 × 10^7^) for 30 minutes prior to being added to the aquaterrarium, and (ii) control – each individual frog was kept in a Petri plate with 10 ml of *Bd*-free distilled water for 30 minutes prior to being added to the aquaterrarium. Our experiment used a total of 116 individual frogs. We fed individual frogs pinhead crickets *ad libitum* during the course of the experiment. At the end of the experiment (day 65), we euthanized all surviving animals with Lidocaine 5% and deposited specimens at Coleção de Anfíbios Célio F. B. Haddad at UNESP, Rio Claro, SP, Brazil.

### Molecular analysis

We swabbed all individual frogs at the moment of capture (swab #1), and ten days after inoculation (swab #2). We swabbed each limb 5 times, specifically between the digits, and also 5 times in each inguinal region^44^. Swab #2 also included dead or dying individuals screened before day 10. We stored each sampling swab in 1.5 ml sterile tubes. We extracted genomic DNA from each swab using Qiagen DNeasy kits. Finally, we diluted 1:10 all samples for qPCR analysis (quantitative polymerase chain reaction) and used standards ranging from 0.1 to 1000 zoospore genome equivalents (g.e.) following procedures described by Boyle *et al*.^45^.

### Statistical analyses

We performed a Generalized Linear Model (GLM) with binomial distribution and logit link to test whether *Bd* prevalence varied between amphibian species with direct development and aquatic larvae. We used Generalized Linear Mixed Models (GLMMs) to test for the effect of host developmental mode (aquatic vs. terrestrial) on infection loads of amphibians exposed to *Bd*. In this model, we included the following explanatory variables: developmental mode as a fixed effect and host species as a random effect. Because a considerable number of individual hosts died (and were swabbed) before day 10 post-inoculation, we also included in the model the day of swab #2 as a fixed effect. For the response variable, we subtracted *Bd* load (log) of swab #1 from swab #2 to estimate a variable accounting for previous exposure in nature (i.e., gain in *Bd* load).

To test for differential mortality rates between *Bd*-inoculated and control treatments for each host species we built mortality curves using survival-reliability analyses. To test whether host life history had an effect on tolerance besides resistance we built a parametric survival model including the following explanatory variables as fixed effects: life history, gain in *Bd* load, the interactive terms life history vs. gain in *Bd* load (because this one-level interaction is a good proxy for tolerance^46^) and survival (time) as the response variable. We also included as a fixed effect in this model the residuals of a parametric survival model testing the effect of species (categorical variable: 6 levels) on host survival. We performed all analyses in JMP Pro v.12 (SAS, 2016).

### Data availability

Full database will be uploaded into DryAd.

### Ethics statement

All experiments were performed in accordance with the university guidelines and regulations for animal care and husbandry. Experimental protocols were approved by Universidade Estadual Paulista (UNESP) and the local animal care committee [Comissão de Ética no Uso de Animal – CEUA (5/2015; #39/2015)].

## Electronic supplementary material

Supplementary information
