# Supplementary material for: Low resistance to chytridiomycosis in direct-developing amphibians
Source: Sci Rep. 2017 Nov 30;7:16605. doi: 10.1038/s41598-017-16425-y (PMC5709405; doi:10.1038/s41598-017-16425-y)
Supplement: Supplementary file 1 — Supplementary information [file 41598_2017_16425_MOESM1_ESM.pdf]

Low resistance to chytridiomycosis in direct-developing amphibians

Andréa F. C. Mesquita[1], Carolina Lambertini[2], Mariana Lyra[1], Leo R. Malagoli[1], Timothy Y. James[3], Luís Felipe Toledo[2], Célio F. B. Haddad[1], C. Guilherme Becker*[4]

[1] Universidade Estadual Paulista (UNESP), Instituto de Biociências, Departamento de Zoologia, and Centro de Aquicultura (CAUNESP), 13506-900 Rio Claro, SP, Brazil

[2] Departamento de Biologia Animal, I.B., Universidade Estadual de Campinas, 13083-862 Campinas, SP, Brazil

[3] Department of Ecology and Evolutionary Biology, University of Michigan, 48109, Ann Arbor, MI, U.S.A.

[4] Department of Biological Sciences, The University of Alabama, 35487 Tuscaloosa, AL, U.S.A.

Corresponding author:
C. Guilherme Becker
*cgbecker1@ua.edu

Supplementary Table S1

Table S1. GLMM testing the effect of host life history on gain in *Bd* load during the experiment. Day of swab included as fixed effect and species included as random effect.

**Parameter**	**d.f. (den)**	**t**	***P***
Intercept	22.73	1.23	0.231
Life history	3.925	-4.3	0.013*
Day of swab	53.55	1.99	0.051

Random effect of species accounted for 47.32%

Supplementary Photo S1

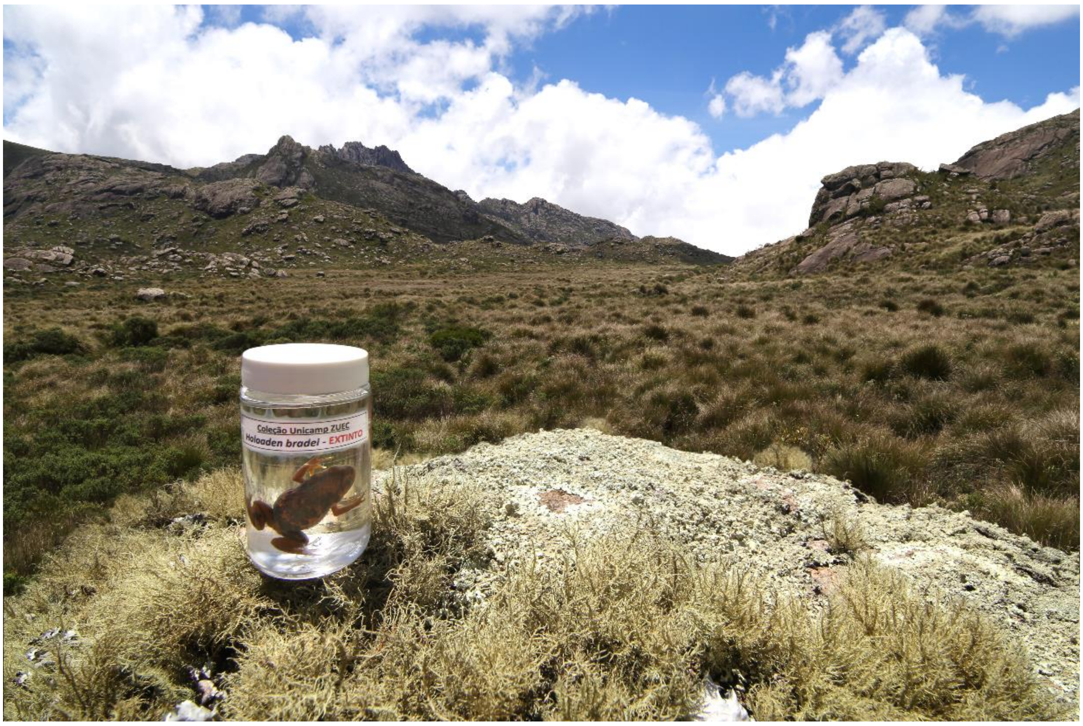

Photo S1. Specimen of the endemic direct-developing frog *Holoaden bradei* (Craugastoridae), last seen in the wild in 1978 and presumably extinct due to chytridiomycosis[18]. Die-offs were never seen at the high elevation quotas of Parque Nacional do Itatiaia, Brazil's Atlantic forest.
